# RAS Dataset: A 3D Cardiac LGE-MRI Dataset for Segmentation of Right Atrial Cavity

**DOI:** 10.1038/s41597-024-03253-9

**Published:** 2024-04-20

**Authors:** Jinwen Zhu, Jieyun Bai, Zihao Zhou, Yaqi Liang, Zhiting Chen, Xiaoming Chen, Xiaoshen Zhang

**Affiliations:** 1 Department of Electronic Engineering, College of Information Science and Technology, Jinan University, Guangzhou, China; 2 Auckland Bioengineering Institute, the University of Auckland, Auckland, New Zealand; 3 Department of Cardiology, The First Affiliated Hospital of Jinan University, Guangzhou, China

**Keywords:** Magnetic resonance imaging, Atrial fibrillation

## Abstract

The current challenge in effectively treating atrial fibrillation (AF) stems from a limited understanding of the intricate structure of the human atria. The objective and quantitative interpretation of the right atrium (RA) in late gadolinium-enhanced magnetic resonance imaging (LGE-MRI) scans relies heavily on its precise segmentation. Leveraging the potential of artificial intelligence (AI) for RA segmentation presents a promising solution. However, the successful implementation of AI in this context necessitates access to a substantial volume of annotated LGE-MRI images for model training. In this paper, we present a comprehensive 3D cardiac dataset comprising 50 high-resolution LGE-MRI scans, each meticulously annotated at the pixel level. The annotation process underwent rigorous standardization through crowdsourcing among a panel of medical experts, ensuring the accuracy and consistency of the annotations. Our dataset represents a significant contribution to the field, providing a valuable resource for advancing RA segmentation methods.

## Background & Summary

Atrial fibrillation (AF) is a globally significant chronic disease, being the most common cardiac arrhythmia, and is associated with substantial morbidity and mortality^1,2^. The suboptimal clinical management of AF largely stems from a fundamental lack of understanding of atrial anatomy^3^. Recent advancements, particularly the widespread use of gadolinium-based contrast agents in assessing atrial fibrosis and scarring through late gadolinium-enhanced magnetic resonance imaging (LGE-MRI)^4^, have significantly improved the visualization of organ structures and related components^5^. Clinical investigations utilizing LGE-MRI in AF patients have highlighted that the extent and distribution of atrial fibrosis can reliably predict the success of ablation procedures^6^. Recent studies using LGE-MRI for atrial assessments have further emphasized the crucial role of atrial structure in comprehending and treating AF^3,7^. Therefore, a direct analysis of atrial structure is vital for effective AF treatment.

Atrial segmentation is a fundamental process involving the extraction of atrial cavity structures from LGE-MRI images. This process serves as a crucial preliminary step in enabling the objective evaluation and quantitative analysis of atrial structure within the context of AF. While extensive research has been conducted on the automatic and semi-automatic segmentation of the left atrium (LA), given its central role in AF studies, it is equally imperative to conduct comprehensive structural assessments of the right atrium (RA)^8,9^. Despite the relatively limited exploration of the pathological changes occurring in the RA within the context of AF, existing evidence strongly suggests that AF exerts its impact on both atria^10^. Therefore, it is imperative to delve into the intricate relationship between AF and the RA. This connection is primarily attributed to a complex interplay of structural, electrical, and metabolic remodeling processes that transpire within the RA^11^. Consequently, research endeavours dedicated to the segmentation of the RA from LGE-MRI scans are indispensable.

Manual segmentation is essential for precise analysis but can be time-consuming and labour-intensive, especially in the context of medical research. To enhance efficiency and accuracy, automated and semi-automated segmentation methods play a crucial role. In the 2018 Left Atrium Segmentation Challenge^12^, 15 teams utilized CNN-based segmentation methods, but 12 proposed CNN designs based on the popular U-Net architecture, achieving outstanding performance. The adoption of the popular U-Net architecture effectively enhanced the effectiveness of atrial segmentation. For instance, D. Borra *et al*.^13^ utilized a CNN-based U-SWNN for 3D left atrium segmentation, achieving a Dice score of 0.911. Xiong *et al*.^14^ employed a dual-branch multi-scale convolutional neural network, significantly improving segmentation results. In contrast, D. Borra *et al*.^15^ proposed a comprehensive two-stage workflow for automatic LA cavity segmentation, involving traditional automated segmentation algorithms for LA localization (first stage) and refined LA segmentation based on CNN outputs (second stage). Although algorithms for the LA are very advanced, there are currently no algorithms for the RA.

However, the development and evaluation of these automated approaches heavily rely on access to extensive datasets with comprehensive annotations. Presently, a noticeable gap exists in dedicated research focused on RA segmentation, and publicly available datasets catering to this specific need are limited. For instance, one available dataset is derived from the 2017 Multi-Modality Whole Heart Segmentation (MM-WHS) challenge^16–18^, which is based on non-contrast MRI scans. While non-contrast MRI yields precise images, contrast MRI, particularly LGE-MRI, offers superior clarity for detecting smaller tissue structures and assessing their extent within the surrounding tissues. LGE-MRI scans have proven invaluable for studying atrial fibrosis^14^. In the field of AF research, several datasets targeting LGE-MRI have been established, such as the 2018 Left Atrial Challenge^12^ and the 2022 Left Atrial and Scar Quantification and Segmentation Challenge^19–21^. However, datasets specifically dedicated to RA segmentation from LGE-MRI scans remain notably absent.

Thus, we introduce the RAS dataset^22^, a valuable resource comprising 50 high-resolution LGE-MRI scans, each with spatial dimensions of either 576 × 576 × 88 or 640 × 640 × 88 pixels. These scans have undergone meticulous pixel-wise manual annotation, performed by four highly trained graduate students and subsequently validated by three experienced advisors. The RAS dataset^22^ represents a significant contribution to the field, serving as a valuable resource for researchers engaged in developing and evaluating automatic RA segmentation algorithms. Furthermore, it has the potential to support the creation of image-based personalized models, thereby advancing our understanding and treatment of AF.

## Methods

### Data collection

The RAS dataset^22^ only provides labels for the right atrium, while the original data belongs to the 2018 Left Atria Challenge^12^ (https://www.cardiacatlas.org/atriaseg2018-challenge/atria-seg-data/) and has been made public. Each 3D MRI patient data in the dataset was acquired using a clinical MRI scanner, specifically a 1.5 Tesla Avanto or 3.0 Tesla Verio whole body MRI scanner. These scans were performed approximately 20–25 months after the injection of gadolinium contrast agent (Multihance, manufactured by Bracco Diagnostics Inc., Princeton, NJ).

### Image annotation

**Fig. 1:**
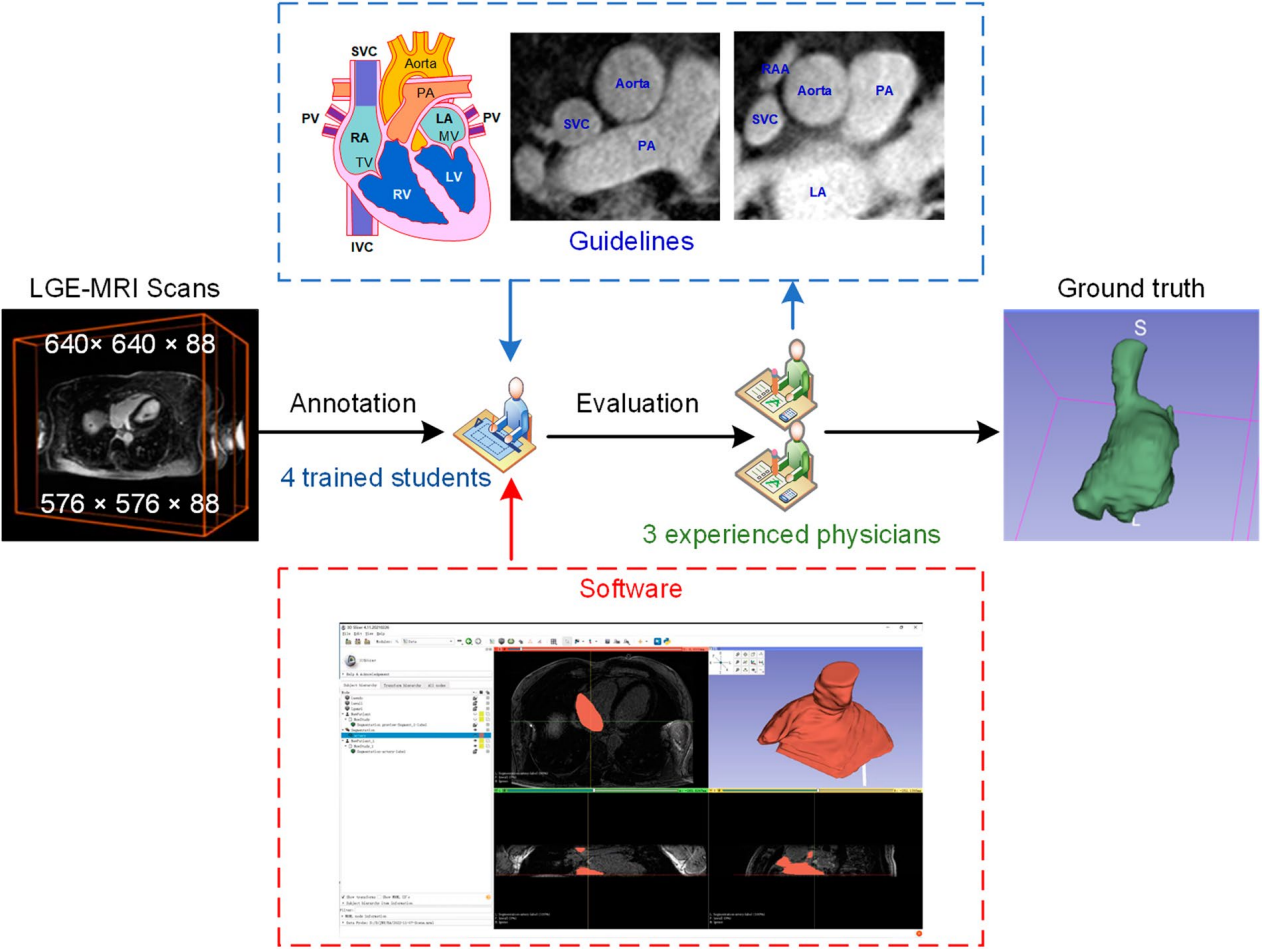
The workflow of image annotation process.

The annotation team consisted of a group of highly qualified individuals, including three experienced physicians and four postgraduates specializing in biomedical-related fields. These annotators underwent comprehensive training, which included online meetings and in-person guidance from the three experienced physicians. The primary focus of this training was to familiarize the annotators with the structure of the RA as it appears in LGE-MRI images. Each trained annotator was responsible for segmenting 25 LGE-MRI images using the pencil tool in Slicer 5.0.3, a software tool available at https://www.slicer.org/. This segmentation process involved pixel-wise annotation, where each pixel was carefully labelled to identify the RA structures, including the tricuspid valve (TV) and right atrial appendages (RAA) as well as the ostia of the superior/inferior vena cava (SVC/IVC). The resulting annotations were subjected to individual evaluations by the physicians. If an annotation was deemed unsatisfactory or inaccurate, the respective image was returned to the student for re-labelling (as illustrated in Fig. 1). During this annotation process, the following points should be followed: (1) ideally, there is a continuous relationship of adjacent pixels in the contour of each two-dimensional image, and there is a hierarchical relationship between the shape and size changes; and (2) Fibrosis in the right atrial wall appears bright white. When there is no obvious white border, the border is determined based on the difference in local gray values. The ground truths are binary, representing the presence or absence of RA structures, and are stored in the Near Raw Raster Data (NRRD) format.

**Fig. 2:**
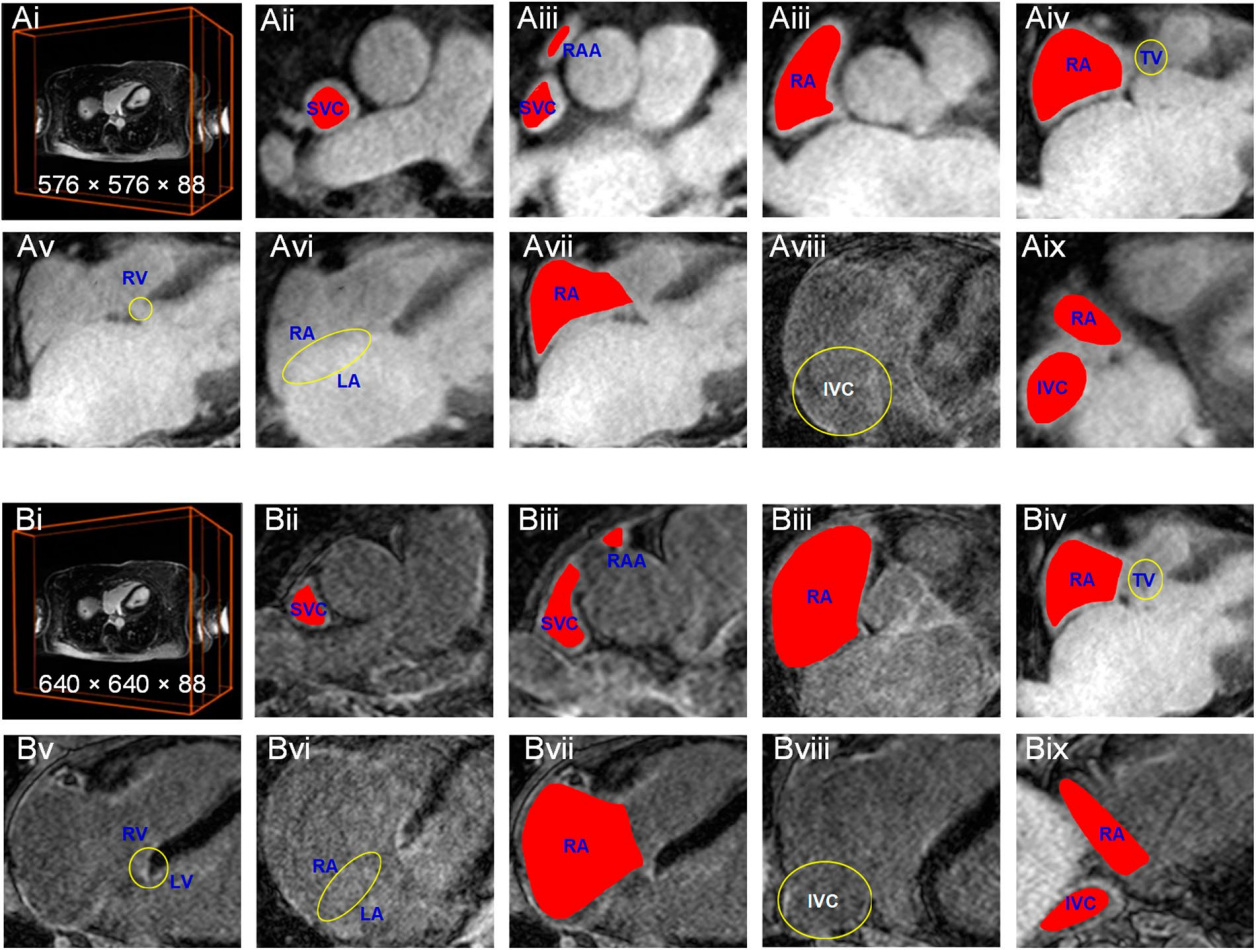
The example process of labelling a 3D LGE-MRI image with the spatial size of 576 × 576 × 88 and 640 × 640 × 88.

**Fig. 3:**
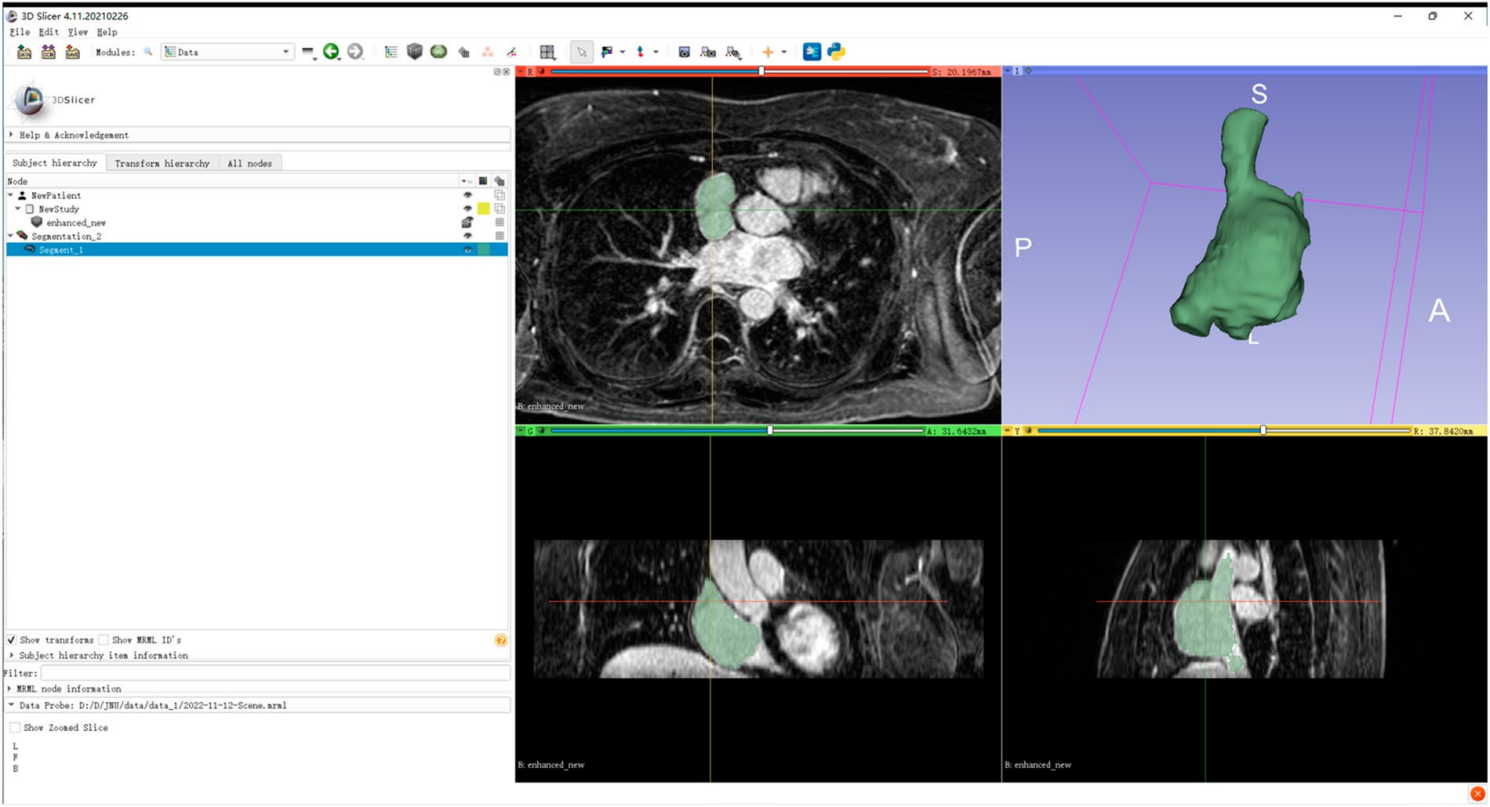
Labelling the RA from the X-, Y- and Z-axis directions with the Slicer 5.0.3.

In the following, we detail the process of labelling a 3D LGE-MRI image with spatial dimensions of either 576 × 576 × 88 (Fig. 2Ai) or 640 × 640 × 88 (Fig. 2Bi). This labelling procedure can be broken down into the following key steps:

- **Step 1 - Identifying SVC Region**: To begin, we observed the SVC region, which typically exhibits a circular or oblate shape. This region was annotated in approximately the first 12 slices (Fig. 2Aii or Fig. 2Bii).
- **Step 2 - Marking RAA Area:** Moving above the SVC area, the RAA, often appearing as a smaller ellipse or triangle, was annotated in the subsequent 4–6 slices (Fig. 2Aiii or Fig. 2Biii).
- **Step 3 - Defining the RA Region:** The RAA area, connected to the SVC region, forms the broader RA region. This region was labeled in approximately the following 30 slices (Fig. 2Aiv or Fig. 2Biv). Throughout the annotation process, we relied on several anatomical landmarks to ensure precise labelling of the RA: a) Tricuspid Valve (TV): Serving as a reference point, the TV helped us delineate the boundary of the RA in specific image slices (e.g., Fig. 2Av or Fig. 2Bv). b) RV-LV Connection: The clear connection between the Right Ventricle (RV) and the Left Ventricle (LV) (e.g., Fig. 2Avi or Fig. 2Bvi) served as a visual guide for accurate RA labelling. c) RA-LA Wall: The wall separating the RA from the LA (e.g., Fig. 2Avii or Fig. 2Bvii) was another vital reference point used for precise identification and labelling of the RA. At this stage, we encountered approximately 25 slices with both RA and RV (e.g., Fig. 2Aviii or Fig. 2Bviii).
- **Step 4 - Transition to IVC Region:** As the LA region gradually disappeared, the IVC region with a circular shape emerged below the RA region. Approximately 15 slices featured both RA and IVC regions (e.g., Fig. 2Aix or Fig. 2Bix). Subsequently, the following slices exclusively featured the IVC region.
- **Step 5 - Fine-Tuning Corrections:** After labelling in the Z-axis direction, we conducted adjustments in the X- and Y-axis directions to ensure the smoothness and continuity of the ground truth (Fig. 3).

## Data Records

All data records^22^ are available as files on the web page 10.5281/zenodo.10781134. In the unzipped folder, the “lgemri.csv” file describes the correspondence between the original LGE-MRI image and its RA cavity label file, and the “ras” folder contains the ground truth label corresponding to the RA cavity. The specific images in the “ras” folder are the ground truth for the corresponding images^12^ (https://www.cardiacatlas.org/atriaseg2018-challenge/atria-seg-data/), and their correspondences are described in the “lgemri.csv” file. Images in the “ras” folder contain pixels labeled 0 and 1, where 0 represents the background and 1 represents the RA cavity .

## Technical Validation

In this study, each LGE-MRI image underwent annotation by one annotator, followed by refinement by a physician. Consequently, inter-annotator consistency warrants investigation. We selected 25 LGE-MRI images from the complete dataset to form an annotation set and assigned these images to two annotators (Dr1 and Dr2). We also evaluated the quality of labels predicted by the classical U-Net model^23^ compared to manual annotations performed by human experts. Dice and Jaccard indices can be used to represent the overlap of validation results, while recall and specificity can indicate the positive-to-negative ratio of validation results. Table 1 displays their respective Dice, Jaccard, recall, and specificity scores, namely AI vs. Dr1, AI vs. Dr2, and Dr1 vs. Dr2. We found that the results between artificial intelligence and humans (AI vs. Dr1 and AI vs. Dr2) were lower than those among humans (Dr1 vs. Dr2), indicating the challenge of automated segmentation for the right atrium. Specifically, for Dr1 vs. Dr2, the average Dice coefficient was calculated to be 93.85%, the Jaccard coefficient was 85.52%, the specificity coefficient was 99.95%, and the recall coefficient was 93.71%, indicating a very close agreement between the annotators.

**Table 1:** Intra-observer variability.

	Dice	Jaccard	recall	specificity
AI vs. Dr1	90.66	83.09	93.63	99.20
AI vs. Dr2	90.47	82.78	92.82	99.16
Dr1 vs. Dr2	93.85	85.92	93.71	99.95

## Usage Notes

Users should cite this paper in their research output and acknowledge the contribution of this dataset in their study.

## Data Availability

No novel code used in the construction of RAS dataset^22^.
